# N_2_O emission associated with shifts of bacterial communities in riparian wetland during the spring thawing periods

**DOI:** 10.1002/ece3.9888

**Published:** 2023-03-08

**Authors:** Xiaoai Cao, Huamin Liu, Yang Liu, Jin Jing, Lu Wen, Zhichao Xu, Xuhua Liu, Dongwei Liu, Yi Zhuo, Lixin Wang

**Affiliations:** ^1^ College of Ecology and Environment Inner Mongolia University Hohhot China; ^2^ Yinshanbeilu Grassland Eco‐hydrology National Observation and Research Station China Institute of Water Resources and Hydropower Research Beijing China; ^3^ Bayannur Sub‐station, Inner Mongolia Environmental Monitoring Station Bayannur China; ^4^ Collaborative Innovation Center for Grassland Ecological Security (Jointly Supported by the Ministry of Education of China and Inner Mongolia Autonomous Region) Hohhot China; ^5^ Ministry of Education Key Laboratory of Ecology and Resource Use of the Mongolian Plateau Hohhot China

**Keywords:** 16S rRNA, Illumina MiSeq sequencing, riparian wetland, soil bacterial community, spring freeze–thaw

## Abstract

Soil freeze–thaw processes lead to high nitrous oxide (N_2_O) emissions and exacerbate the greenhouse effect. The wetlands of the Inner Mongolia Plateau are in the pronounced seasonal freeze–thaw zone, but the effect of spring thaw on N_2_O emissions and related microbial mechanisms is still unclear. We investigated the effects of different periods (freeze, freeze–thaw, and thaw) on soil bacterial community diversity and composition and greenhouse gas emissions during the spring freeze–thaw in the XiLin River riparian wetlands in China by amplicon sequencing and static dark box methods. The results showed that the freeze–thaw periods predominantly impact on the diversity and composition of the bacterial communities. The phyla composition of the soil bacteria communities of the three periods is similar in level, with *Proteobacteria*, *Chloroflexi*, *Actinobacteria*, and *Acidobacteria* dominating the microbial communities. The alpha‐diversity of bacterial communities in different periods varies that the freezing period is higher than that of the freeze–thaw period (*p* < .05). Soil total carbon, soil water content, and microbial biomass carbon were the primary factors regulating the abundance and compositions of the bacterial communities during spring thawing periods. Based on functional predictions, the relative abundance of nitrification and denitrification genes was higher in the freezing period than in the thawing period, while the abundance was lowest in the freeze–thawing period. The correlation results found that N_2_O emissions were significantly correlated with amoA and amoB in nitrification genes, indicating that nitrification may be the main process of N_2_O production during spring thaw. This study reveals potential microbial mechanisms of N_2_O emission during spring thaw and provides data support and theoretical basis for further insight into the mechanism of N_2_O emission during spring thaw.

## Abstract 2

This study highlights the difference in soil bacterial communities and N cycle functional genes in different spring thawing periods, and the microbial mechanism of N_2_O during the spring thaw, which is helpful to better understand the microbial process of N_2_O emission under climate change.

## 1 INTRODUCTION

Soil freezing–thawing in spring is a common natural phenomenon in the mid‐high‐latitude regions, which significantly contributes to soil nitrous oxide (N_2_O) (Chen et al., 2018; Song et al., 2019). Globally, the spring thaw increases N_2_O emissions from soils by nearly 150% (Gao et al., 2018). N_2_O is crucial in atmospheric photochemical processes that can destroy stratospheric ozone and have a global warming potential that is 265 times that of carbon dioxide (Bahram et al., 2022). Large N_2_O emissions during spring thawing have received increasing attention in agricultural soil (Chen et al., 2018), forest soil (Flesch et al., 2018), and steppe soil (Wang et al., 2017; Zedong et al., 2015). The N_2_O emissions during the spring thaw are mainly due to the fact that N_2_O produced in deep soils during the freezing period will accumulate in large quantities under the permafrost layer, and when the frozen layer at the soil surface dissipates, the accumulated N_2_O will be released in large quantities with increased diffusivity (Goldberg et al., 2009; Wagner‐Riddle et al., 2017). Moreover, freeze–thaw cycles increase nutrient availability (Gao et al., 2019), change microbial community structure (Liu et al., 2022), and microorganisms, as mediators of the material cycle and energy transfer, play an important role in promoting the release of soil N_2_O (Song et al., 2019; Wagner‐Riddle et al., 2017). However, the microbial dynamic processes associated with large N_2_O emissions during spring melt have not been well studied.

Freeze–thaw is the main driving force affecting soil microbial activity. It has been reported that freeze–thaw can change microbial community structure (Ren et al., 2018) and microbial substrate utilization patterns (Han et al., 2018). On the one hand, soil freezing reduces the effectiveness of soil water content (SWC) and increases soil osmotic pressure, which leads to the death of vulnerable microorganisms and thus affects the biomass and composition of microbial communities (Koponena & Bååth, 2016; Ren et al., 2018; Walker, 2010). On the other hand, freeze–thaw leads to the breakdown of soil aggregates and litter decomposition after the death of microorganisms, releasing a large amount of effective nutrients, while ice and snow melting promotes the formation of surface active layers, further enhancing the viability of resurrected microorganisms (Schuur et al., 2009). As a buffer zone between terrestrial and aquatic ecosystems, riparian wetlands are more susceptible to freeze–thaw cycles due to their periodic flooding. However, the microbial changes and key driving factors during the spring thaw period need to be further studied.

The Xilin River basin in China is a typical semi‐arid steppe‐type inland river, and its riparian zone wetlands are an important part of the regional ecosystem, which has an irreplaceable role in regulating climate, water retention, and the carbon and nitrogen cycling (Gou et al., 2015). The Xilin River basin is located in a frequent freeze–thaw zone, which is a unique natural laboratory for exploring the relationship between freeze–thaw and riparian biogeochemistry. In our experimental sites, N_2_O fluxes are associated with freeze–thaw and usually occur from the middle of March to the end of April, when temperatures rose above 0°C (Cao et al., 2022). Most previous studies have been conducted indoors to explore N_2_O emission bursts under controlled conditions and the soil physicochemical driving mechanisms of N_2_O emissions, with little exploration of the microbial mechanisms of N_2_O emissions during freeze–thaw.

In this study, the soil bacterial community in XiLin River riparian wetland during spring thaw was analyzed by amplicon sequencing. Our aims were (i) to investigate how soil bacterial community properties and N_2_O emission fluxes respond to spring thawing; (ii) to study the influencing factors of soil bacterial community changes in spring thawing period; and (iii) to explore how soil bacterial community characteristics impact N_2_O emissions dynamics during spring thawing.

## 2 MATERIALS AND METHODS

### 2.1 Site description and sampling

**FIGURE 1:**
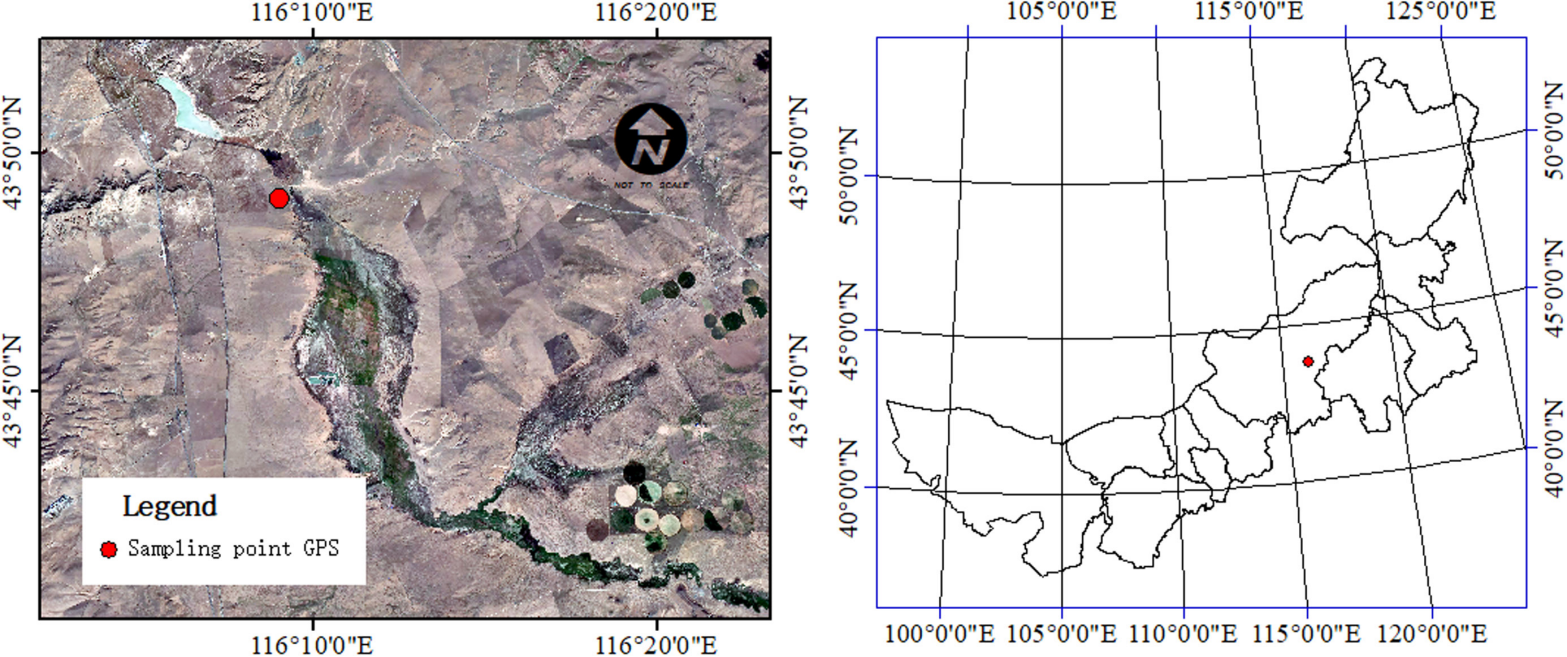
The geographical location map of the study area.

The study area is located in the seasonal freeze–thaw zone (116°9′1″E, 43°49′2″N) of the XiLin River Basin in Inner Mongolia, China (Figure 1). It is a temperate continental grassland climate. The average temperature was −22°C in January and 18.3°C in July. There are many freeze–thaw processes from mid‐late October to mid‐late November in autumn and from mid‐late March to mid‐late May in spring.

From the edge to the center of the riparian, the vegetation changed from *Carex appendiculata* to *Phragmites australis* with the increase of standing water depth. Therefore, this study selected *Ph. australis* and *Ca. appendiculata* communities for investigation. Three 1 m × 1 m plots (representing three replicates) were selected at random in each plant community in February 2018, to remove 0–2 cm of fresh dead fallen material from the soil surface within the plot and smashed into steel bases at fixed points to ensure as much consistency as possible in the experiment; there were 30 m between each plant community. The freeze–thaw period was divided into three phases based on the variation of soil temperature: freezing (March 2018), freeze–thaw (April 2018), and thawing (May 2018). A total of 18 soil samples (5–10 cm) were collected around the base of the plot in typical weather during the three periods for microbial community characteristics and soil chemical analysis. Soil pH was measured as a 2.5:1 water‐soil slurry using a standard pH meter. Soil moisture content was determined using the drying method, where soil samples were dried at 105°C for 24 h. Soil nitrate nitrogen (${\mathrm{NH}}_{4}^{+}-N$) and ammonia nitrogen (${\mathrm{NO}}_{3}^{-}-N$) were determined using a continuous flow analyzer (FIAstar 5000, Foss Tecator). Soil total carbon (TC) and total nitrogen (TN) were determined by the elemental analyzer. Chloroform fumigation extraction was used to evaluate microbial biomass carbon (MBC) and microbial biomass nitrogen (MBN). The moist soil were divided into two parts, fumigated soil (chloroform fumigation for 24 h at 25°C) and nonfumigated soil, extracted with 0.5 M potassium sulfate (K_2_SO_4_) by shaking for 30 min, The organic carbon content of the fumigated and nonfumigated extracts was determined by TOC (SKALAR Primacs SLC TOC), and the ammoniacal nitrogen content of the extracts was determined by AA3 Continuous flow analyzer and the calculation methods of MBC and MBN are the same. It is dividing the difference between the extracted carbon or nitrogen content in the fumigated and nonfumigated soil by a correction factor of 0.45 (Rotbart et al., 2017). N_2_O sampling was performed using the static dark chambers and Picarro G 2308. N_2_O sampling methods and flux calculations are detailed in the supplementary (Cao et al., 2022).

### 2.2 DNA extraction, amplification, and sequencing

Microbial community genomic DNA was extracted from soil samples using the E.Z.N.A.® soil DNA Kit (Omega Bio‐Tek) according to the manufacturer's instructions. The DNA extract was checked on 1% agarose gel, and DNA concentration and purity were determined with NanoDrop 2000 UV–vis spectrophotometer (Thermo Scientific). The hypervariable region V3‐V4 of the bacterial 16 S rRNA gene was amplified with primer pairs 338F (5′‐ACTCCTACGGGAGGCAGCAG‐3′) and 806R (5′‐GGACTACHVGGGTWTCTAAT‐ 3′) by an ABI GeneAmp® 9700 PCR thermocycler (ABI). The PCR 130 amplification of 16 S rRNA gene was performed as follows: initial denaturation at 95°C for 3 min, followed by 27 cycles of denaturing at 95°C for 30 s, annealing at 55°C for 30 s and extension at 72°C for 45 s, and single extension at 72°C for 10 min, and end at 4°C. The PCR mixtures contain 5 × TransStart FastPfu buffer 4 μL, 2.5 mM dNTPs 2 μL, forward primer (5 μM) 0.8 μL, reverse primer (5 μM) 0.8 μL, TransStart FastPfu DNA Polymerase 0.4 μL, template DNA 10 ng, and finally ddH_2_O up to 20 μL. PCR reactions were performed in triplicate. The PCR product was extracted from 2% agarose gel and purified using the AxyPrep DNA Gel Extraction Kit (Axygen Biosciences) according to the manufacturer's instructions and quantified using Quantus™ Fluorometer (Promega).

Purified amplicons were pooled in equimolar and paired‐end sequenced (2 × 300) on an Illumina MiSeq platform (Illumina) according to the standard protocols by Majorbio Bio‐Pharm Technology Co. Ltd.

### 2.3 Sequence data preprocessing

The raw 16 S rRNA gene sequencing reads were demultiplexed, quality‐filtered by Trimmomatic, and merged by FLASH with the following criteria: (i) the 300 bp reads were truncated at any site receiving an average quality score of <20 over a 50 bp sliding window, and the truncated reads shorter than 50 bp were discarded, reads containing ambiguous characters were also discarded; (ii) only overlapping sequences longer than 10 bp were assembled according to their overlapped sequence. The maximum mismatch ratio of the overlap region is 0.2. Reads that could not be assembled were discarded; (iii) Samples were distinguished according to the barcode and primers, and the sequence direction was adjusted, exact barcode matching, 2 nucleotide mismatch in primer matching.

UPARSE (version 7.1, http://drive5.com/uparse/) was used to cluster operational taxonomic units (OTUs) using a 97% similarity criterion (Edgar et al., 2013), and chimeric sequences were discovered and eliminated. RDP Classifier (http://rdp.cme.msu.edu/) was used to compare the taxonomy of each OTU representative sequence to the 16S rRNA database, with a confidence level of 0.7.

After loading the normalized bacterial OTUs table into PICRUSt2, the bacterial nitrification and denitrification functions were predicted using the KEGG database (Douglas et al., 2020). The weighted closest sequenced taxon index (NSTI) scores were used to confirm the accuracy of PICRUSt2 predictions for each sample. The NSTI score is <0.17 (Langille et al., 2013).

### 2.4 Statistical analysis

We averaged all datasets of two plant communities in order to analyze the overall differences between different freeze–thaw periods. To establish the significance of differences, a one‐way analysis of variance (ANOVA) with the Tukey's multiple comparison post hoc test was used, and a value of 0.05 was considered statistically significant. The Vegan program was used to do a nonmetric multidimensional scaling (NMDS) ordination to demonstrate the grouping of bacterial community composition change based on the Bray–Curtis distance of OTUs. RDA and variation partitioning were used to investigate the association between soil microbial community structure and environmental conditions. Environmental factors were selected by vif.cca, and the environmental factors with vif >10 were removed from the RDA analysis. The R package rdacca. hp was used to decompose the explanatory rate of each explanatory variable, and a negative single explanatory variable indicated that this variable had a negligible role in explaining the response variables and the sum of all the variables eventually gives 100%. The Pearson correlation coefficient between N_2_O emission and soil parameters, as well as the top 10 prevalent bacterial phyla and soil properties, were determined and exhibited on a heat map. All analyses were performed using R version 4.2.2.

## 3 RESULTS

### 3.1 N_2_O flux in spring freeze–thaw periods

**FIGURE 2:**
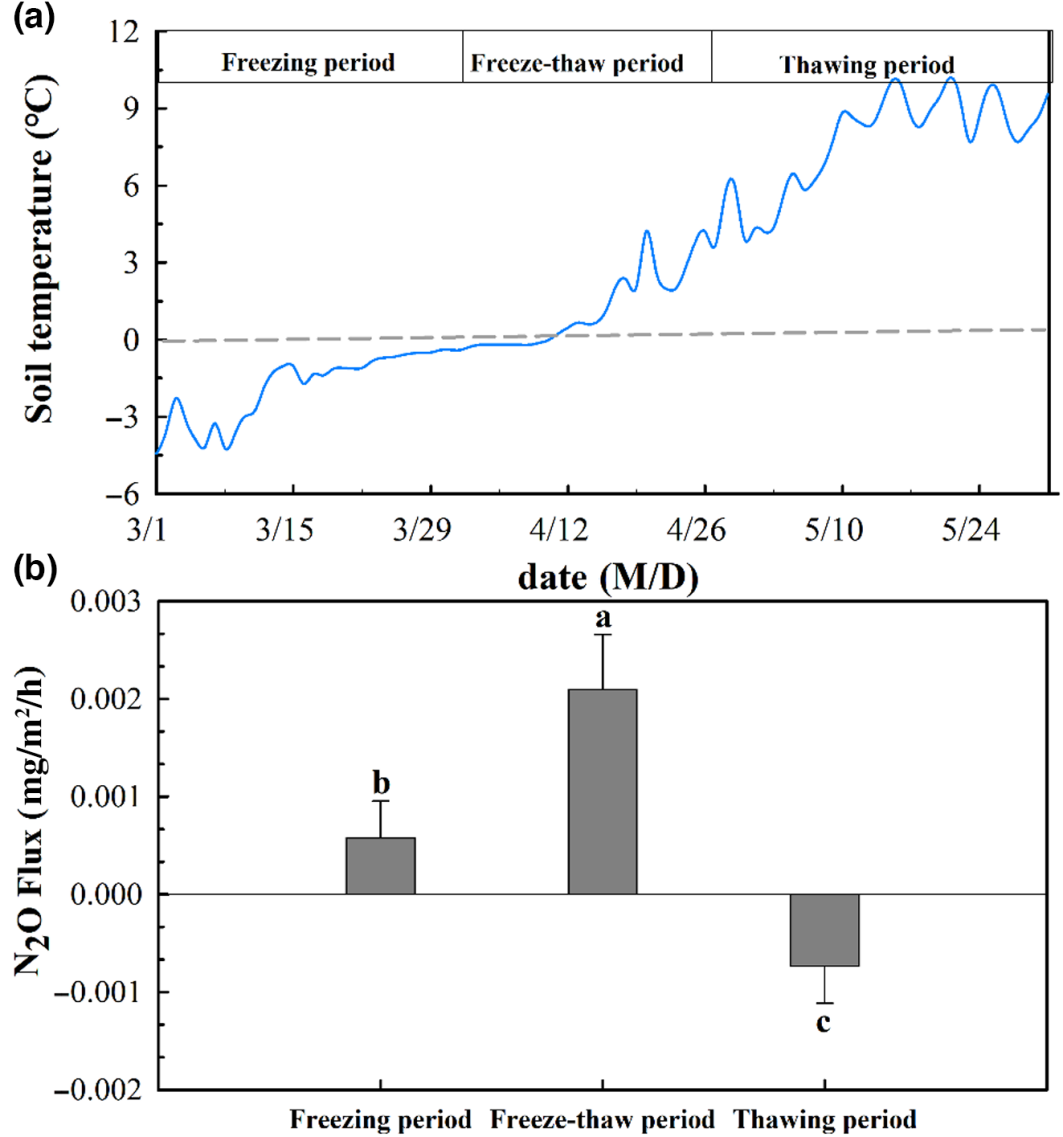
(a) Soil temperature changes in riparian wetlands during spring freeze–thaw; (b) N_2_O emission fluxes during different freeze–thaw periods.

The spring freeze–thaw period is divided into the freezing period, freeze–thaw period, and thawing period based on the change in soil temperature. During most of the freezing period, the soil temperature in the sample plots was below 0°C; after sampling during the freezing period, the soil temperature began to increase and fluctuate around 0°C. The final sampling was conducted when the daily freezing and thawing frequency was high; sampling during the thawing period was conducted when the soil temperature remained stable above 5°C (Figure 2a). Riparian wetlands soil still had a small amount of N_2_O emission during the freezing period, and large emission of N_2_O was observed during the freezing–thawing period; N_2_O was absorbed after thawing (Figure 2b). The variation of soil pH among the periods was low and similar. Soils were weakly alkaline. With the increase in soil temperature, SWC decreased significantly (Table S1). Soil ${\mathrm{NO}}_{3}^{-}-N$ was significantly higher during freeze–thaw than during freezing (*p* < .05), while the contents of soil TC, TN, MBC, MBN, and ${\mathrm{NH}}_{4}^{+}-N$ did not change significantly. Correlation analyses showed that N_2_O emissions were significantly negatively correlated with soil temperature and positively correlated with SWC, TC, MBC, ${\mathrm{NH}}_{4}^{+}-N$, and ${\mathrm{NO}}_{3}^{-}-N$ (Figure S1).

### 3.2 The bacterial diversity and community composition in spring freeze–thaw periods

**FIGURE 3:**
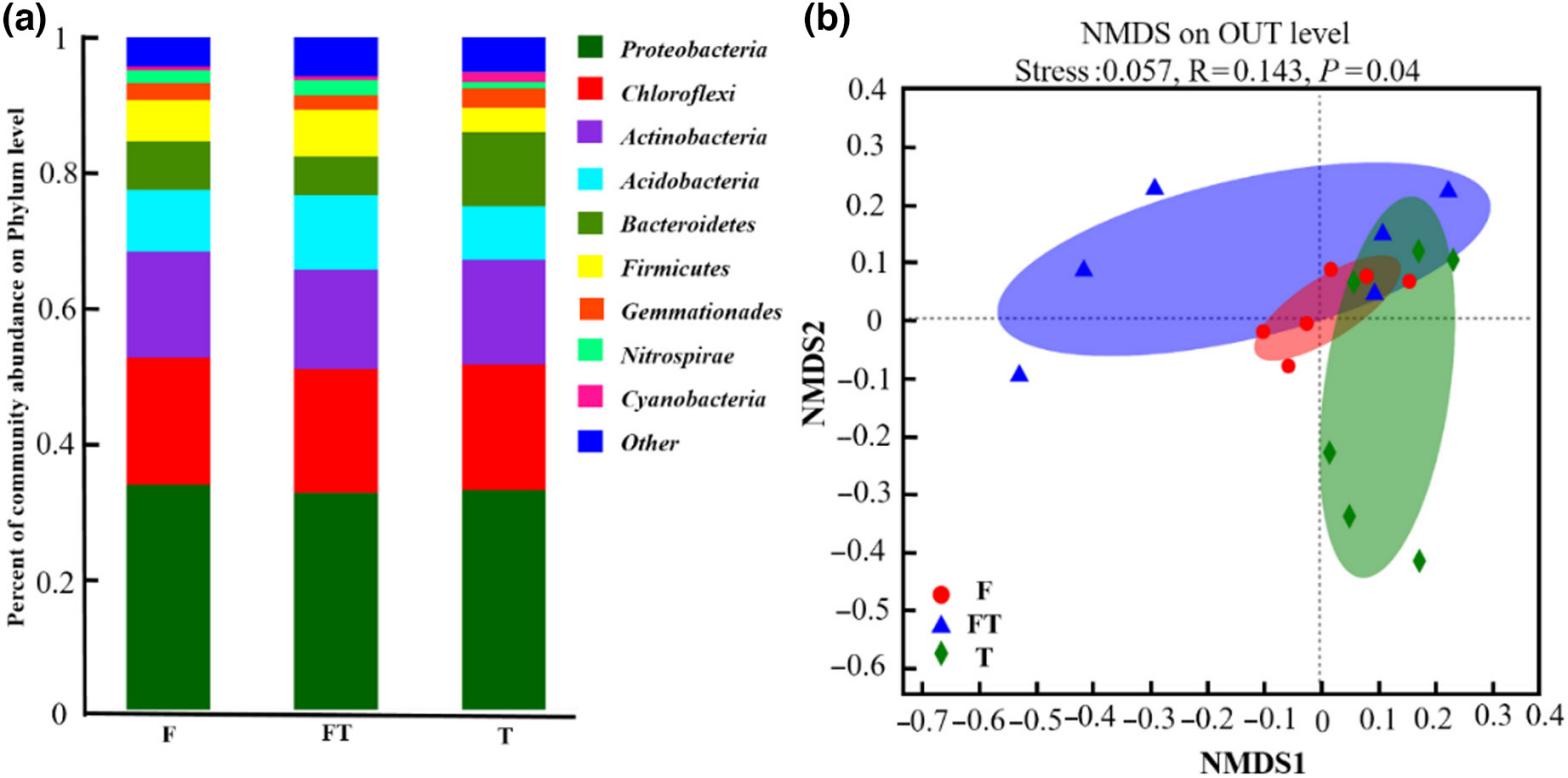
(a) Relative abundance of dominant bacterial (at phylum level) taxa at freeze, freeze–thaw, and thaw periods (F is freezing period, FT is freezing and thawing period, T is thawing period. The same below); (b) NMDS ordination based on Bray–Curtis similarities of bacterial communities at freeze, freeze–thaw, and thaw periods.

We obtained a total of 937,019 high‐quality 16 S rRNA gene sequences from 18 samples (2 communities × 3 periods × 3 replicates). The rarefaction curves showed that our sequencing depth sufficiently captured the diversity of the bacterial population (Figure S2). The most abundant bacterial phylum across all samples was *Proteobacteria* (33.10% on average) followed by *Chloroflexi* (18.53%), *Actinobacteria* (15.27%), *Acidobacteria* (9.37%), *Bacteroidetes* (7.94%), *Firmicutes* (5.57%), *Gemmatimonadetes* (2.51%), *Nitrospirae* (1.67%), and *Cyanobacteria* (0.86%) (Figure 3a). The relative abundance of *Proteobacteria*, which was the most dominant taxa in the three periods. *Chloroflexi* did not change significantly during the three periods. *Acidobacteria* has the highest fraction in freeze–thaw periods but has not achieved significant change (Figure 3a). The relative abundance of *Firmicutes*, *Pacubacteria*, and *Armatimonadetes* was significantly higher during the freeze–thaw period than during the freezing period (*p* < .05) (Figure S3).

Nonmetric multidimensional scaling analysis based on Bray–Curtis similarity distance revealed that the three periods had no significant influence on bacterial community structure (Figure 3b). Alpha‐diversity showed that the highest diversity was observed in the freezing period whereas lowest was in the freeze–thaw period (Table 1).

**TABLE 1:** Alpha‐diversity of soil bacterial communities in riparian wetland during freeze–thaw period.

Period	Shannon	Chao	Heip	Coverage
F	6.78 ± 0.13	3575.40 ± 134.96	0.30 ± 0.03	0.98 ± 0.00
FT	6.51 ± 0.51	3136.40 ± 474.89	0.28 ± 0.09	0.98 ± 0.00
T	6.63 ± 0.31	3202.70 ± 450.02	0.29 ± 0.05	0.98 ± 0.00

*Note*: F, freezing period; FT, freezing and thawing period; T, thawing period. Lowercase letters indicate significant differences at *p* < .05 (Student's *t*‐test).

**FIGURE 4:**
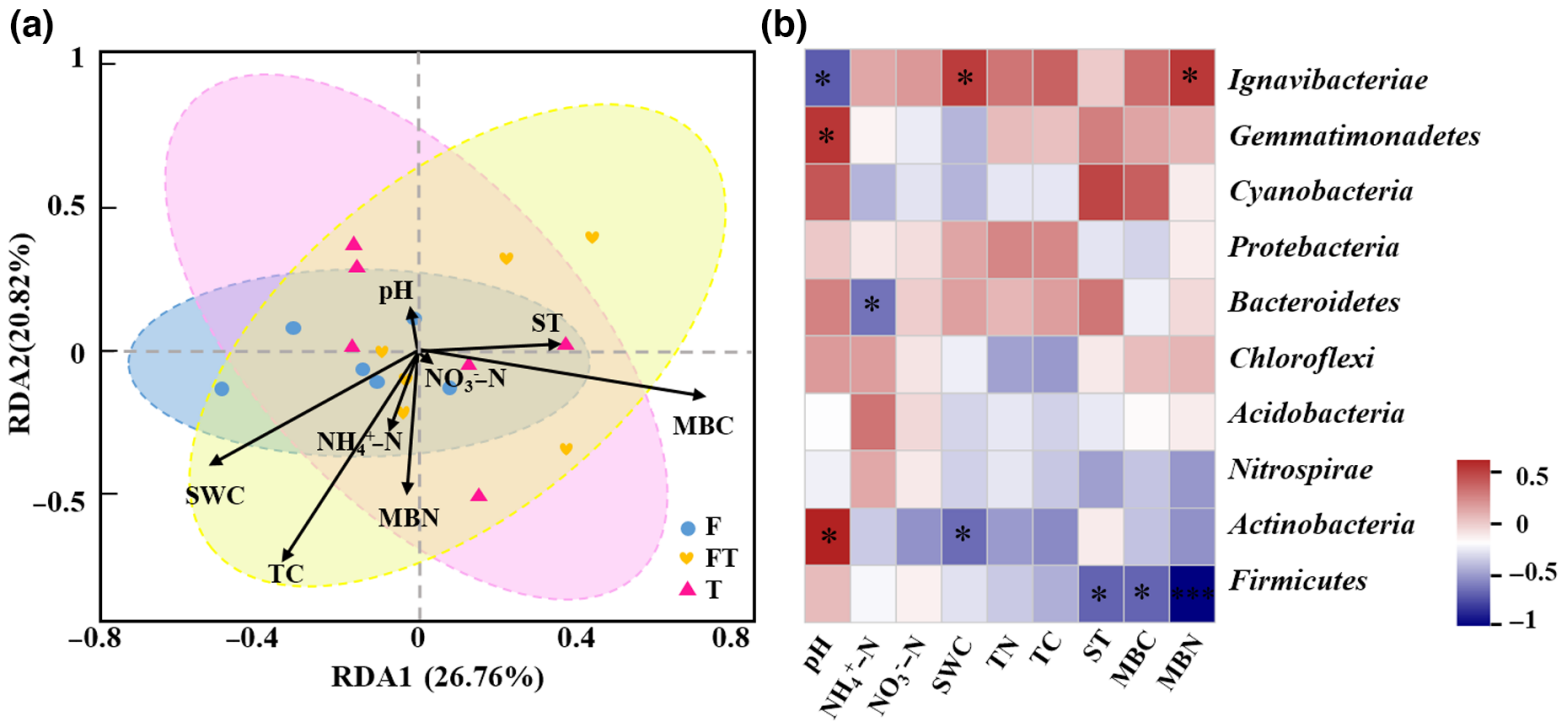
(a) RDA analysis between samples and soil physicochemical factors; (b) Heatmap of correlations between dominant phyla and soil physicochemical factors. MBC, microbial biomass carbon; MBN, microbial biomass nitrogen; ${\mathrm{NH}}_{4}^{+}-N$, ammonia nitrogen; ${\mathrm{NO}}_{3}^{-}-N$, nitrate nitrogen; ST, soil temperature; SWC, soil water content; TC, total carbon; TN, total nitrogen.

Redundancy analysis (RDA) was used to examine the association between bacterial community structure and soil parameters, and the eight environments variables (i.e., ST, SWC, pH, TC, ${\mathrm{NH}}_{4}^{+}-N$, ${\mathrm{NO}}_{3}^{-}-N$, MBC, and MBN) explained 47.58% of the total changes in the composition of soil bacterial communities (Figure 4a). TC, MBC, and SWC explained 116.96%, 64.20%, and 30.87% of the differences in bacterial community structure, respectively (Figure 4a, Figure S4). The correlation heatmap found that *Firmicutes* was significantly negatively correlated with ST, MBC, and MBN (*p* < .05) (Figure 4b). *Actinobacteria* was significantly positively correlated with pH but significantly negatively correlated with SWC (*p* < .05). Bacteroidetes was significantly negatively correlated with ${\mathrm{NH}}_{4}^{+}-N$. *Gemmatimonadetes* was significantly positively correlated with pH (*p* < .05); *Ignacibacteriae* was significantly negatively correlated with SWC and MBN but significantly positively correlated with pH (*p* < .05) (Figure 4b).

### 3.3 Relationships between N_2_O emission and nitrogen cycling gene and environmental factors

**FIGURE 5:**
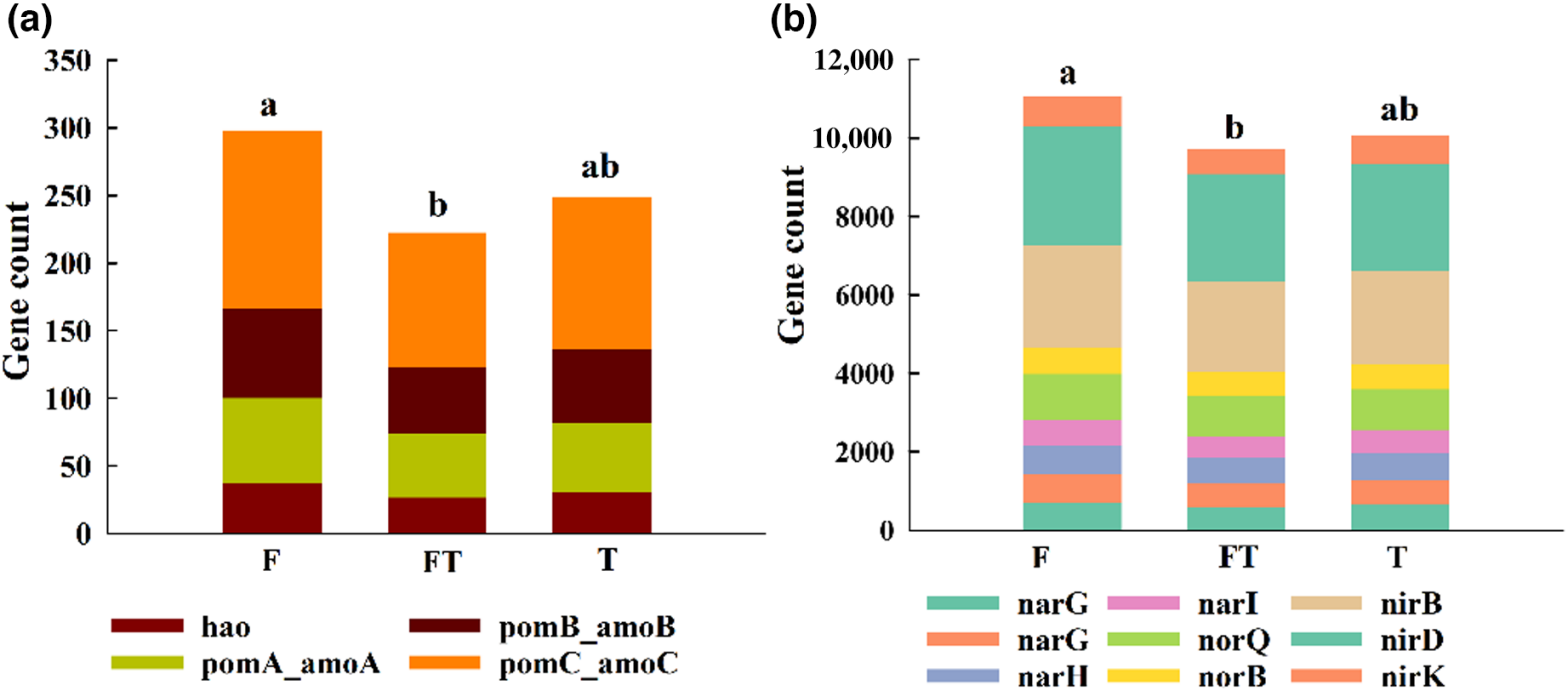
Prediction of bacterial functional genes involved in nitrification (a) and denitrification (b). Lowercase letters indicate significant differences from one another at *p* < .05 (Tukey–Kramer test).

PICRUSt2 was used to estimate the relative abundance of bacterial functional genes involved in the nitrification and denitrification processes (Figure 5). The relative abundance of nitrification genes and denitrification genes in soil was significantly affected by three periods. Soil nitrification genes were significantly higher in the freezing period than in the freeze–thaw period (*p* < .05) (Figure 5a), and denitrification genes were also the most abundant in the freezing period (Figure 5b). Correlation analysis showed that N_2_O emission was significantly negatively correlated with nitrification genes amoA and amoB but not with other nitrogen‐cycling genes (Table 2).

**TABLE 2:** Correlation between N_2_O emission and nitrification and denitrification genes abundance.

	N_2_O	narG	narH	narI	norQ	nirB	nirD	nirK	Hao	amoA	amoB	amoC
N_2_O	1	−0.261	−0.133	−0.206	−0.317	0.290	−0.235	0.072	−0.233	−0.672*	−0.672*	−0.654
narG	−0.261	1	0.970**	0.945**	0.283	0.529	0.247	0.199	0.482	0.522	0.522	0.298
narH	−0.133	0.970**	1	0.910**	0.391	0.676*	0.384	0.400	0.598	0.555	0.555	0.345
narI	−0.206	0.945**	0.910**	1	0.189	0.535	0.061	0.111	0.288	0.463	0.463	0.231
norQ	−0.317	0.283	0.391	0.189	1	0.294	0.596	0.727*	0.675*	0.719*	0.719*	0.606
nirB	0.290	0.529	0.676*	0.535	0.294	1	0.570	0.730*	0.430	0.300	0.300	0.098
nirD	−0.235	0.247	0.384	0.061	0.596	0.570	1	0.863**	0.761*	0.605	0.605	0.601
nirK	0.072	0.199	0.400	0.111	0.727*	0.730*	0.863**	1	0.688*	0.548	0.548	0.459
hao	−0.233	0.482	0.598	0.288	0.675*	0.430	0.761*	0.688*	1	0.747*	0.747*	0.741*
amoA	−0.672*	0.522	0.555	0.463	0.719*	0.300	0.605	0.548	0.747*	1	1.000**	0.872**
amoB	−0.672*	0.522	0.555	0.463	0.719*	0.300	0.605	0.548	0.747*	1.000**	1	0.872**
amoC	−0.654	0.298	0.345	0.231	0.606	0.098	0.601	0.459	0.741*	0.872**	0.872**	1

*p < .05, **p < .01.

## 4 DISCUSSION

### 4.1 Effects of spring freeze–thaw on soil bacterial community structure in riparian wetland

High‐latitude wetland ecosystems are susceptible to freeze–thaw cycles, resulting in changes in soil bacterial composition and diversity (Ji, Liu, et al., 2022). The results of this study showed that although the relative abundance of some bacterial members was changed by the spring freeze–thaw cycle, the composition of soil bacteria was not completely altered (Figure 3a). Since soils that are naturally exposed to cold and changing environmental conditions may be hardy and resilient bacterial species that selected a stable bacterial community that is only little affected by freeze–thaw (Ji, Kong, et al., 2022; Makhalanyane et al., 2016). Furthermore, the relative insensitivity of bacteria to freeze–thaw cycles may be due to the fact that the nutrients of deceased microorganisms can sustain the development of surviving bacteria following a freeze–thaw cycle (Chapman et al., 2017; Xue et al., 2020). The dominant phylum in the three periods of spring melting was *Proteobacteria*, *Chloroflexi*, and *Actinobacteria*, and they accounted for 66.9% of the total abundance. This result was similar to previous studies on Arctic water‐logged permafrost (Hultman et al., 2015; Juan et al., 2018). Our analyses revealed that *Proteobacteria* had the largest relative abundance, which was owed to its flexibility (Jiang et al., 2013; Kang et al., 2021). In addition, *Chloroflexi* and *Actinobacteria* were predominant in the freeze–thaw period, indicating their ability to withstand low temperatures and limited nutrient availability (Chapman et al., 2017; Xue et al., 2020). Although the *Proteobacteria*, *Chloroflexi*, and *Actinobacteria* are the most numerically abundant, *Firmicutes*, *Pacubacteria*, and *Armatimonadetes* were also very important in spring freeze–thaw periods, and their abundance was significantly higher in freeze–thaw periods than in freezing periods (Figure S3). *Firmicutes* include spore‐forming groups like Clostridia, which are not only adapted to be active in sub‐zero temperatures (Hultman et al., 2015) but can also resist environmental disturbances (Paredes‐Sabja et al., 2010).

The alpha‐diversity of soil bacteria was altered by spring freeze–thaw cycles. In our study, the bacterial community alpha‐diversity in the freezing period was higher than that in the freeze–thaw period. The relatively high alpha‐diversity in the freeze period may be because of the adaptation of surviving bacteria to frozen conditions (Lim et al., 2019). Winter bacterial communities may primarily use complex and recalcitrant substrates (i.e., cellulose and salicylates) to resist frost stress. Thus, different functional responses to environmental stress may lead to the segregation of bacterial communities (Robroek et al., 2013). However, bacterial diversity decreased during the freeze–thaw period, which suggested that spring freeze–thaw events in soil may potentially select bacterial taxa which can withstand freeze–thaw stress and eliminate those that cannot (Lim et al., 2019; Walker et al., 2006). Studies in high arctic confirmed that the soil bacterial community diversity decreased after freeze–thaw (Lim et al., 2019; Liu et al., 2022). We observed no significant changes in bacterial beta diversity between freeze–thaw periods, which is consistent with some studies (Ren et al., 2018). However, some studies found that freeze–thaw cycles altered bacterial community structure (Li et al., 2022), which may be due to differences in freeze–thaw intensity and frequency in indoor simulations. The RDA results showed that TC, MBC, and SWC were important explanatory factors affecting bacterial community (Figure 4a). There were no significant changes in TC and MBC content throughout the spring freeze–thaw period (Table S1). In addition, SWC has been thought to be an important factor in mediating soil bacterial communities (Banerjee et al., 2016; Keet et al., 2019). Han et al. (2017) concluded that under conditions of high SWC, the extracellular water of microorganisms freezes and freezing stress causes intracellular dehydration, thus maintaining the balance of osmotic pressure inside and outside the cell, which is lower than freezing stress and thus has no major effect on microorganisms (Han et al., 2017). In this study, the freezing period had the highest soil moisture (Table S1), which may have protected the bacterial community structure from being disrupted, thus leaving the beta diversity unchanged.

### 4.2 The influence factors of N_2_O emission during spring freeze–thaw

We observed that the soil N_2_O emissions increased significantly during the spring freeze–thaw period, similar to previous investigations (Cui et al., 2016; Song et al., 2019; Wang et al., 2013). The reason for this might be that N_2_O was created in the unfrozen subsoil and then physically liberated from the soil surface as the frozen soil thawed (Teepe et al., 2001; Wager‐Riddle et al., 2007). Our results showed that the relative abundance of denitrification genes was significantly higher in the freezing period than in the freeze–thaw period (Figure 5b). This indicates that when the soil temperature is below 0°C, a large number of denitrification genes produced a considerable amount of N_2_O and sequester it in the soil, releasing it as the soil melts. On the other hand, freeze–thaw disrupts the soil bulk structure (Oztas & Fayetorbay, 2003), causing soil microbial cells to break down and release mineral nitrogen (Judd et al., 2010), increasing the content of ${\mathrm{NO}}_{3}^{-}-N$ (Table S1) and supplied adequate substrate for N_2_O producers, leading to an increase in N_2_O emission. Because of the restricted substrate availability during the ensuing thawing phase, soil N_2_O production decreased dramatically and stayed almost constant. This was also observed in other terrestrial habitats (Kariyapperuma et al., 2011; Stefanie et al., 2010; Zedong et al., 2015).

The complicated metabolic processes of nitrification and denitrification in soil, which depend on the availability of oxygen and inorganic nitrogen (${\mathrm{NH}}_{4}^{+}$ and ${\mathrm{NO}}_{3}^{-}$), release N_2_O as an intermediate product (Braker & Conrad, 2011; Malla et al., 2005; Zhu et al., 2013). Our results revealed that the relative abundance of soil nitrobacteria gene and denitrobacteria gene was high during the spring freeze–thaw period (Figure 5), and soil N_2_O emissions were driven by soil soluble nitrogen (${\mathrm{NH}}_{4}^{+}-N$ and ${\mathrm{NO}}_{3}^{-}-N$) (Figure S1). These results suggested that microbial nitrification and denitrification may be the cause of N_2_O emissions during the spring freeze–thaw cycles (Smith et al., 2010; Song et al., 2019). ŠImek and Cooper (2002) discovered that the pH threshold for limiting N_2_O emissions pathways was around 4.40 because denitrification became the primary source of N_2_O emissions at lower pH values. The pH value in our study was between 8.2 and 8.7 during the spring freeze–thaw periods (Table S1), and correlation analysis results showed that N_2_O was significantly correlated with nitrification genes (Table 2), indicating that nitrification was the main source of N_2_O emission during spring thaw, which was inconsistent with other research results (Mørkved et al., 2006; Wagner‐Riddle et al., 2008). This may be related to ${\mathrm{NH}}_{4}^{+}-N$ and ${\mathrm{NO}}_{3}^{-}-N$ content in the three periods (Table S1). ${\mathrm{NH}}_{4}^{+}-N$ and ${\mathrm{NO}}_{3}^{-}-N$ are the substrates of nitrifying bacteria and denitrifying bacteria, respectively, which affect the rate of nitrification and denitrification. During three periods of spring thawing, the content of ${\mathrm{NH}}_{4}^{+}-N$ is higher than that of ${\mathrm{NO}}_{3}^{-}-N$, which provides a more sufficient substrate for nitrifying bacteria and promotes the nitrifying rate to produce N_2_O.

## 5 CONCLUSION

We investigated the composition of soil bacterial and the relative abundances of nitrification and denitrification genes in the riparian wetland during spring freeze–thaw cycles. Spring freeze–thaw did not significantly change the soil bacterial composition, but the relative abundance and diversity of some groups within the bacterial community varied. We also individuated important soil physicochemical properties affecting these microbial changes. Our result indicates that a large amount of N_2_O emission during the spring freeze–thaw period potentially results from soil nitrification.

## AUTHOR CONTRIBUTIONS

**Xiaoai Cao:** Data curation (equal); formal analysis (lead); investigation (lead); methodology (lead); writing – original draft (lead). **Huamin Liu:** Conceptualization (supporting); visualization (supporting); writing – original draft (supporting); writing – review and editing (supporting). **Yang Liu:** Data curation (supporting); resources (supporting); supervision (supporting); validation (supporting); writing – review and editing (supporting). **Jin Jing:** Resources (supporting); software (supporting); supervision (supporting); writing – review and editing (supporting). **Lu Wen:** Data curation (supporting); methodology (supporting); validation (supporting); visualization (supporting); writing – review and editing (supporting). **Zhichao Xu:** Data curation (supporting); investigation (supporting); methodology (supporting); software (supporting). **Xuhua Liu:** Investigation (supporting); methodology (supporting); writing – original draft (supporting). **Dongwei Liu:** Formal analysis (supporting); funding acquisition (supporting); resources (supporting); software (supporting). **Yi Zhuo:** Software (supporting); supervision (supporting); writing – review and editing (supporting). **Lixin Wang:** Conceptualization (lead); data curation (lead); investigation (supporting); methodology (lead); project administration (lead); writing – review and editing (lead).

## Supporting information


Appendix S1

## Data Availability

DNA sequences: NCBI: BioProject IDPRJNA905183. http://www.ncbi.nlm.nih.gov/bioproject/905183.
