# Supplementary material for: N2O emission associated with shifts of bacterial communities in riparian wetland during the spring thawing periods
Source: Ecol Evol. 2023 Mar 8;13(3):e9888. doi: 10.1002/ece3.9888 (PMC9994613; doi:10.1002/ece3.9888)
Supplement: Supplementary file 1 — Appendix S1 [file ECE3-13-e9888-s001.docx]

Research methods supplementary material

**N_2_O flux measurements**

Gas flux measurements were conducted by applying static dark chambers and Picarro G 2308. Five stainless steel bases (50 cm×50 cm×25 cm) with a water groove on top were installed in the soil before sampling and were kept throughout the experimental period. Gas samples were taken between 8:00 am and 12:00 am. During sampling, an open-bottom stainless steel chamber (50 cm×50 cm ×50 cm, equipped with two fans) was placed over the base and filled with water in the groove to ensure tightness. The stainless steel chamber was wrapped with a foam plate to ensure that the temperature in the chamber did not change much during sampling. The sample chamber was equipped with a thermometer, a balance tube, and a triple-valve. Air sample inside the chamber was taken every 10 min over a 30-min period by using a 60 ml plastic syringe in the bags (total of four samples). The air temperature inside the chamber and atmospheric pressure were measured during sampling. Corrections were made for air temperature and pressure. Data that did not deviate significantly from linearity were used (*R^2^*>0.90). The gas flux was calculated with the following formula:

$$F=H\times\frac{M}{V_{0}}\times\frac{T_{0}}{T}\times\frac{P}{P_{0}}\times\frac{dC_{t}}{dt}$$

where *F* is the measured gas discharge flux; *H* is the height of the sampling box (m); *M* (g/mol) is the molar mass of the gas; *V_0_*, *T_0_*, *P_0_* are respectively the molars of the gas under standard conditions (22.4 L/mol), air thermodynamic temperature (273.15 K) and air pressure (1.013×10^5^ Pa); *T* and *P* are the thermodynamic temperatures at sampling and the gas pressure at the sampling point; *C*_t_ is the gas concentration (ppm); dC_t_/dt is the slope of gas concentration changing with time at the time of sampling.

**Supplementary Figures**


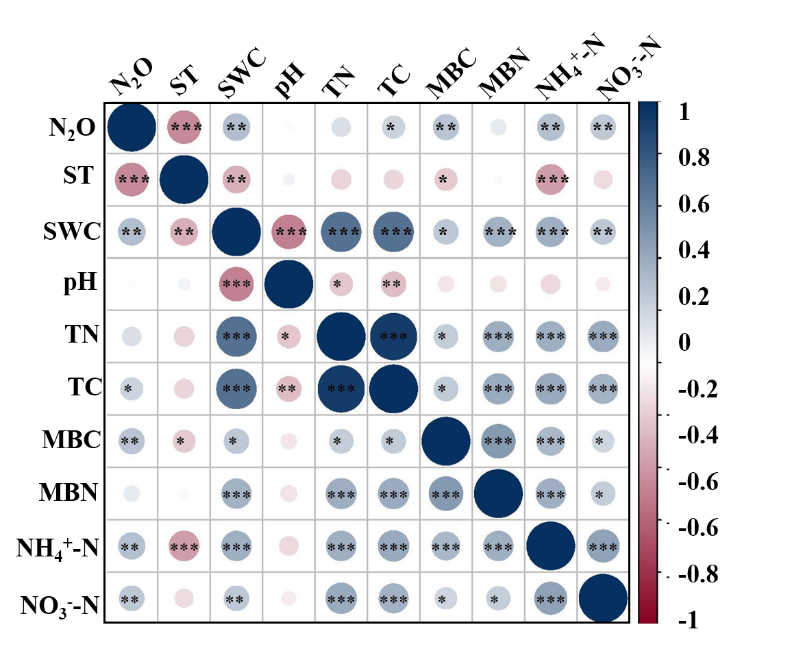


**Fig. S1** Heat map of correlation between N_2_O emission and soil environmental factors

Notes: ST is soil temperature, SWC is soil water content, TC is Total Carbon, TN is Total Nitrogen, MBC is Microbial biomass carbon, MBN is Microbial biomass nitrogen, NH_4_^+^-N is ammonia nitrogen, NO_3_^-^-N is nitrate nitrogen , F is freezing period, FT is freezing and thawing period, T is thawing period (The same below).


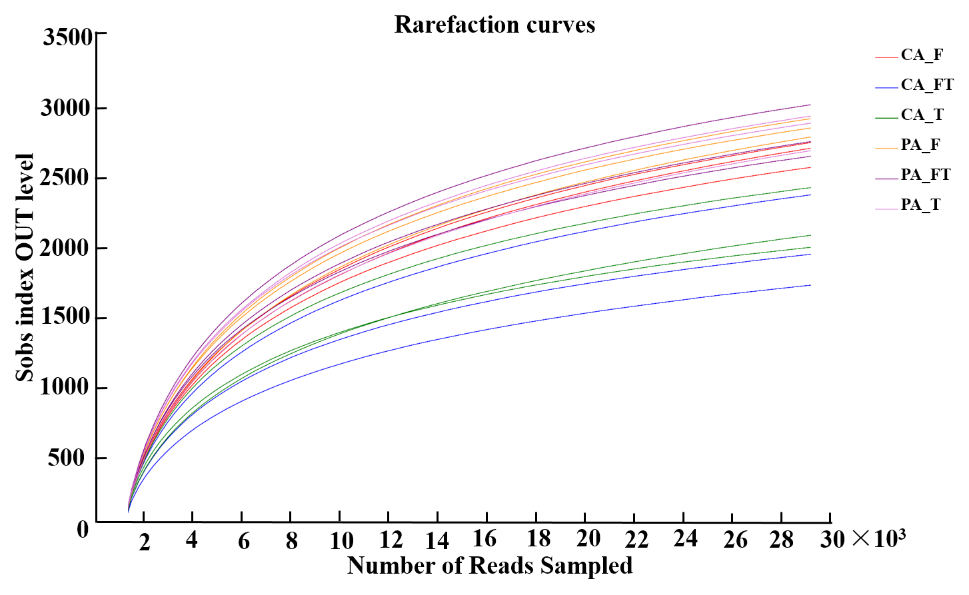


**Fig. S2** Rarefaction curves from non-rarefied data from the bacterial communities in different periods.


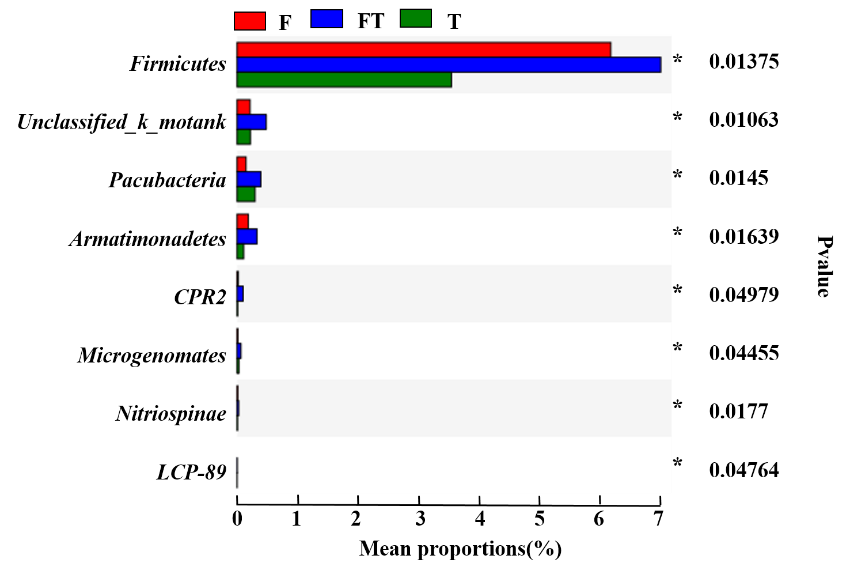


**Fig. S3** Bacterial species composition varied at the phylum level during different periods


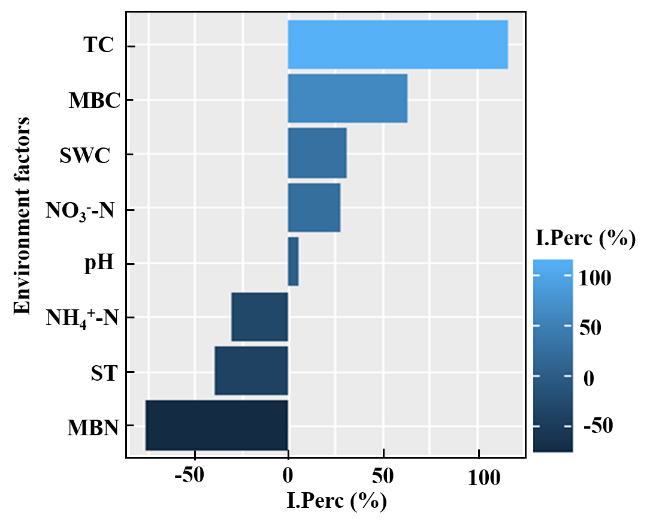


**Fig.S4** Relative variance contribution of environmental factors to the soil bacterial community

**Supplementary Tables**

**Table S1**. Environmental variables from the different periods of riparian wetland.

| Parameter | F | FT | T |
| --- | --- | --- | --- |
| ST (℃) | -0.66±0.33c | 3.72±0.21b | 9.35±0.70a |
| SWC (%) | 38.91±2.66a | 29.29±2.92b | 28.44±2.71b |
| pH | 8.52±0.23a | 8.29±0.07a | 8.45±0.20a |
| TN (mg/g) | 2.97±0.57a | 2.23±0.35a | 2.33±0.41a |
| TC (mg/g) | 36.50±7.11a | 28.05±4.82a | 31.53±5.21a |
| MBC (mg/g) | 540.35±57.42a | 556.14±79.24a | 685.91±95.86a |
| MBN (mg/g) | 40.26±3.65b | 26.31±8.47b | 72.30±15.29a |
| NH_4_^+^-N (mg/g) | 13.48±4.51a | 12.94±1.69a | 10.95±1.93a |
| NO_3_^-^-N (mg/g) | 3.38±1.46b | 11.74±3.93a | 7.19±1.47ab |

Notes: All data are represented by the mean ± sd, significant differences are marked with different letters in each column (*p* < 0. 05).
